# Stress influenced increase in phenolic content and radical scavenging capacity of *Rhodotorula glutinis* CCY 20-2-26

**DOI:** 10.1007/s13205-012-0069-1

**Published:** 2012-05-30

**Authors:** Raj Kumar Salar, Milan Certik, Vlasta Brezova, Marta Brlejova, Vladimira Hanusova, Emília Breierová

**Affiliations:** 1 Department of Biotechnology, Chaudhary Devi Lal University, Sirsa, 125 055 India; 2 Faculty of Chemical and Food Technology, Slovak University of Technology, Radlinskeho 9, 812 37 Bratislava, Slovak Republic; 3 Institute of Chemistry, Slovak Academy of Sciences, Dúbravská cesta 9, 845 38 Bratislava, Slovak Republic

**Keywords:** *Rhodotorula*, Phenolics, Radical scavenging capacity, HPLC, EPR/spin trapping

## Abstract

*Rhodotorula glutinis* CCY 20-2-26 when grown under controlled stress of either NaCl (1–5 %) or H_2_O_2_ (1–5 mM) on basal media exhibited a twofold increase in its total phenolic contents. The radical scavenging capacities (RSCs) as determined by ABTS test were found to be highest in 4 mM H_2_O_2_ (1.44 mM TEAC mg^−1^) and 4 % NaCl (1.13 mM TEAC mg^−1^) as compared to control samples (0.41 mM TEAC mg^−1^). Similarly, the RSCs as determined by DPPH test were also highest in 4 % NaCl (1.83 mM TEAC mg^−1^) and 4 mM H_2_O_2_ (1.78 mM TEAC mg^−1^) compared to control (0.48 TEAC mg^−1^). The relative RSCs from EPR spin-trapping assay for H_2_O_2_-stressed cultures were highest in 1 mM H_2_O_2_ (56.1 μM TEAC g^−1^) whereas in NaCl-stressed cultures it was highest in 5 % NaCl (44.6 μM TEAC g^−1^) as compared to control (30.9 μM TEAC g^−1^). Five phenolic compounds (gallic acid, benzoic acid, catechin, caffeic acid and ferulic acid) were detected for the first time in *R. glutinis* CCY 20-2-26.

## Introduction

Stress in biological tissues is known to bring about a biochemical response involving an increase in the levels of various antioxidant compounds or in the activity of enzymes responsible for the regeneration of antioxidant metabolites (Ramotar et al. 1998). Earlier, several studies were carried out to show that yeast species produce increased levels of carotenoids when they are grown under unfavourable conditions (Certik and Breierova 2002; Marova et al. 2004). Although, yeast is a non-photosynthetic microorganism, there are yeasts that can biosynthesize carotenoids and phenolic compounds in the cell. Carotenoids and phenolic compounds combat various types of cancer and other diseases because of their free radical scavenging and/or provitamin A (carotene) potential (Bhosale and Gadre 2001). Free radicals are known to be a product of normal metabolism. These are also involved in organism’s vital activities including phagocytosis, regulation of cell proliferation, intracellular signalling and synthesis of biologically active compounds (Halliwell 1989; Miquel and Romano-Bosca 2004).

Phenolic compounds from plant kingdom are well documented for their antioxidant properties and health promoting benefits. However, little attention has been paid to the phenolic compounds evaluation in yeasts (Rizzo et al. 2006). In the recent past, both eukaryotic and aerobic prokaryotic organisms have been developed with an overall antioxidant defence system for mitigating the damaging effects of free radicals (Kullisaar 2002; Daeschel 2004; Jaehrig et al. 2008; Chen et al. 2010). Nevertheless, all aerobic organisms including humans have antioxidant defences that protect against oxidative damage and repair damaged molecules. However, the natural antioxidant mechanisms can be inadequate, the supply of antioxidants through dietary ingredients is of great interest for the food industry (Scalbert and Williamson 2000; Greenwald et al. 2001; Chen et al. 2010).

Although, the yeasts have received extensive concern on using them as starter cultures for development of new products (Wouters et al. 2002) and potential probiotics (Psomas et al. 2001, 2003; Kumura et al. 2004), studies related to radical scavenging capacities (RSCs) of yeasts are scanty (Rapta et al. 2005). In order to survive in adverse environment, microorganisms have developed efficient adaptation mechanisms to tide over undesirable stress by activated synthesis of biomolecules (Estruch 2000; Certik and Breierova 2002). Thus, studies related to exogenous stress and scavenging property could explain in part the mechanism of protection against harmful effects of the environment. The purpose of the present investigation was to determine total phenolic compounds of *Rhodotorula glutinis* CCY 20-2-26 grown under controlled stress of NaCl or H_2_O_2_ including its intact cells and cell free extracts. Radical scavenging capacity of its extracts modulated by NaCl or H_2_O_2_ was evaluated by EPR spin-trapping technique, DPPH and ABTS assays which may provide evidence for exploring novel products with antioxidant activity. Further, phenolic compounds were identified using HPLC.

## Materials and methods

### Microorganism and culture conditions

*Rhodotorula glutinis* CCY 20-2-26 was obtained from Culture Collection of Yeasts (CCY, Institute of Chemistry, Slovak Academy of Sciences, Bratislava, Slovak Republic) and maintained on agar slants at 4 °C. It was cultivated on basal medium consisting of (g L^−1^): yeast extract, 5; glucose, 20; (NH_4_)_2_SO_4_, 10; KH_2_PO_4_, 1; K_2_HPO_4_·3H_2_O, 0.2; NaCl, 0.1; CaCl_2_, 0.1; MgSO_4_·7H_2_O, 0.5; and 0.25 mL of microelement solution [(mg L^−1^): H_3_BO_4_, 1.25; CuSO_4_·5H_2_O, 0.1; KI, 0.25; MnSO_4_·5H_2_O, 1; FeCl_3_·6H_2_O, 0.5; (NH_4_)_2_Mo_7_O_24_·4H_2_O, 0.5; and ZnSO_4_·7H_2_O, 1]. For the present study, *R. glutinis* was grown under non-lethal and maximally tolerated concentration of either NaCl (1–5 %) or H_2_O_2_ (1–5 mM). The inoculum (10 % v/v) consisted of 48-h-old cells of *R. glutinis* grown in the above basal media. The cultures were cultivated in 500 mL Erlenmeyer flasks containing 150 mL of cultivation medium on a rotary shaker (140 rpm) at 28 °C to early stationary growth phase. Cells were harvested by centrifugation at 3,000 rpm and washed thrice with distilled water and stored at −20 °C until further analysis.

### Extraction of phenolic compounds

The extraction of phenolic compounds was performed directly on the microbial biomass. Dry yeast biomass (DCW—dry cell weight) was prepared gravimetrically. For preparing methanol extracts, 200 mg of DCW was suspended in 20 mL of methanol in 100-mL conical flasks. The samples were shaken for 10 min and then centrifuged at 5,000 rpm for 10 min. The supernatants were collected. The DMSO extracts were prepared using yeast biomass with 1 mL DMSO (Merck, Germany) at 60 °C for 60 min in 2-mL Eppendorf tubes and then centrifuged at 5,000 rpm for 10 min. The extracts obtained were filtered through membrane filters (0.22 μm). All extracts were stored at −20 °C until further analysis of total phenolic content and RSC.

### Total polyphenolic content determination

Total polyphenol contents were determined on the biomass of harvested cells (herein after called “cells”) and DMSO and methanol extracts (herein after called “extracts”) of *R. glutinis* using Folin–Ciocalteu (FC) reagent following Yu et al. (2004) and Commission Regulation (EEC) No. 2676/90 (1990) with slight modifications. Briefly, 10 mg of yeast biomass was taken and suspended in 10 mL volumetric flask with 0.5 mL of distilled water. Then 0.5 mL of FC reagent (Merck) and 1.5 mL of aqueous sodium carbonate anhydrous solution 20 % (w/v) was added. The flask was filled with distilled water to volume and the suspension was poured off into a centrifuge tube. After 120 min, the suspension was centrifuged at 4,000 rpm for 5 min. The absorbance was read at 765 nm, subtracting the value of a control solution consisting of distilled water instead of biomass. For determination of total polyphenol content in DMSO and methanol extracts, 100 μL of extracts were taken instead of yeast biomass. The amount of total polyphenol was calculated as gallic acid equivalents (GAE) from the standard calibration curve of gallic acid (Sigma-Aldrich) and expressed as milligram gallic acid equivalents per gram of yeast. It should be noted that no significant polyphenolic activity was observed in methanol extracts and thus, only DMSO extracts were used for analysis of RSC.

### DPPH radical scavenging assay

The free RSC of different fractions was measured by the DPPH (1,1-diphenyl-2-picrylhydrazyl) scavenging method according to Yen and Chen (1995) with some modifications. Briefly, 200 μL of DMSO extract was taken in spectrophotometric cell and then 3 mL of 100 μM DPPH (Sigma-Aldrich) (4 mg DPPH in 100 mL methanol) was added. In the reference sample, 200 μl of DMSO was used instead of extracts. The changes in absorbance at 519 nm in minute 10 relative to the reference sample were recorded using a Implen NanoPhotometer 1890 (version, 7122 V2.0.0). The DPPH RSCs were expressed as trolox equivalent antioxidant capacity (TEAC) in μmol g^−1^ of yeast biomass.

### ABTS^•+^ radical cation depolarization assay

Antioxidant activity was measured using a modified method of Re et al. (1999) and Arts et al. (2004). 2,2′-Azino-bis (3-ethylbenzothiazoline-6-sulfonate) (ABTS, Sigma) was used for production of the corresponding radical cation (ABTS^•+^) by dissolving 17.2 mg ABTS and 3.3 mg K_2_S_2_O_8_ (Aldrich) in 5-mL distilled water, and the resulting solution was left to stand for 16 h in dark at room temperature. A stock solution of ABTS^•+^ was prepared by mixing 1 mL of this reaction mixture with 60-mL water. The concentration of ABTS^•+^ was determined by UV–Vis spectroscopy using the characteristic value of molar absorption coefficient at 732 nm, 1*.*5 × 10^4^ mol^−1^ L cm^−1^. After rigorously mixing 2.3-mL ABTS^•+^ solution with 200 μL of DMSO extracts, the UV–Vis spectra were taken in 0.5 s intervals for 10 min using UV-3600 UV–Vis spectrophotometer (Shimadzu, Japan, 1-cm square quartz cell). UV-Vis spectrum of initial ABTS^•+^ solution measured against distilled water was taken as a reference spectrum. The difference in the absorbance in 10th min at 732 nm relative to reference spectrum was used to calculate the antioxidant activity. The ability of samples to eliminate ABTS^•+^ is expressed using 6-hydroxy-2,5,7,8-tetramethylchroman-2-carboxylic acid (trolox, Aldrich) as reference antioxidant and the results were expressed as TEAC in μmol g^−1^ of yeast biomass (DCW).

### EPR spin-trapping technique

The thermal decomposition of potassium persulfate (K_2_S_2_O_8_) in DMSO at 333 K was used as a source of reactive radicals. To measure the RSC of yeast extracts, the EPR spin-trapping technique (Rapta et al. 2005) was used, employing 5,5-dimethyl-1-pyrroline *N*-oxide (DMPO, Sigma-Aldrich) as a spin trap. Sulfate radical anions (SO_4_^•−^) generated upon thermal decomposition of K_2_S_2_O_8_ represent reactive species with high reduction potential, capable to react with a variety of organic compounds (Wardman 1989). In DMSO solvent these paramagnetic species are added to the double bond of DMPO spin-trapping agent producing the corresponding spin adducts (Zalibera et al. 2009). All EPR measurements were carried out in a 4-mm flat quartz cell in a Bruker TE_102_ (ER 4102 ST) cavity using the EMX EPR spectrometer (Bruker, Rheinstetten, Germany) working in the X-band. The ER 4111 VT temperature unit (Bruker, Germany) was used for temperature regulation. The reaction mixture consisted of 100 μL of DMSO extracts (pure DMSO in reference), 100 μL DMSO, 25 μL of 0.2 M DMPO dissolved in DMSO and 25 μL of 0.01 M K_2_S_2_O_8_ (DMSO). A time course of EPR spectra of the DMPO spin adducts was recorded in 66-s intervals for 22 min at 333 K (each spectrum represents an accumulation of three scans). The integral EPR intensity (double integral) found after 22 min of thermal treatment for the sample solution was compared with the reference measurement. The difference between the integral EPR intensities of the reference and the samples in 22nd min characterises the amount of radicals scavenged by the various components present in the sample acting as radical scavengers. The RSC values were calculated as a percentage of scavenged radicals relative to the reference sample (DMSO). These values were recalculated to TEAC using calibration curve measured analogously for trolox solutions in K_2_S_2_O_8_/DMPO/DMSO systems, and so obtained radical scavenging characteristics of investigated samples were evaluated in μmol of trolox/1 g of extract.

### HPLC analysis of phenolic compounds

Phenolic compounds were analysed by HPLC on an Agilent 1100 series HPLC unit with computer-controlled software and system controller. Mobile phase consisted of acetonitrile (**A**) and water/acetic acid (pH 2.8) (**B**). Linear gradient from 5 to 100 % **A** in 35 min and flow rate 1 mL min^−1^ was applied. An autoinjector was used to inject 10 μL of extracts or standards into the HPLC system. Absorbance data were recorded at 272 nm over a period of 35 min. Phenolic compounds’ identification was achieved by the absorbance recorded in the chromatograms relative to external standards. Standards used included gallic acid, benzoic acid, catechin, caffeic acid and ferulic acid. The data were further quantified by ChemStation software B 01 03 (Agilent Technologies).

## Results and discussion

### Production of total phenolic compounds under different culture conditions

In an earlier study conducted by Rapta et al. (2005) increased free radical scavenging and antioxidant activities of metabolites (carotenoids) produced by yeasts under heavy metal stress was reported. However, it is important to know whether only carotenoids are responsible for this increase or some other metabolites, particularly phenolic compounds are also involved. As phenolic compounds are well known for their antioxidant activities, in the present investigation total phenolic contents (TPC) of *R. glutinis* were determined directly on the “cells” as well as from the “extracts”. To the best of our knowledge, this is the first report of occurrence of phenolic compounds in yeasts, particularly in the investigated species.

The extracts of *R. glutinis* cells were prepared in DMSO to observe the effect of stress factors on phenolic contents and antioxidant activities. Under control culture conditions with no medium supplementation with stress factors, TPC produced by *R. glutinis* were 30.8 and 37.69 mg GAE g^−1^ in “cells” and “extracts”, respectively. A substantial increase in TPC was observed when *R. glutinis* was grown under non-lethal and maximally tolerated concentration of either NaCl (1–5 %) or H_2_O_2_ (1–5 mM) on basal media. The phenolic contents as determined directly on the “cells” ranged from 38.22 to 62.72 mg GAE g^−1^ in cultures stressed with different molar concentrations of H_2_O_2_ compared to control (30.8 mg GAE g^−1^). However, it ranged from 18.92 to 40.45 mg GAE g^−1^ in cultures stressed with different concentrations of NaCl (Table 1). On the contrary, the phenolic contents from “extracts” ranged from 34.94 to 47.49 mg GAE g^−1^ in NaCl-stressed cultures whereas it ranged from 29.04 to 50.15 mg GAE g^−1^ in H_2_O_2_-stressed cultures (Table 1). The highest levels of TPC were observed in 4 mM H_2_O_2_ (50.15 mg GAE g^−1^) and 1 % NaCl (47.49 mg GAE g^−1^) -stressed cultures compared to control (37.69 mg GAE g^−1^). The increase in phenolic contents in *R. glutinis* reflects operation of some defence mechanism under stress.

**Table 1:** Total phenolic content of cells and cell free DMSO extracts of *Rhodotorula glutinis* grown under stress of NaCl or H_2_O_2_

Stress factor	Total phenolic contents (GAE mg g^−1^) DCW	
	Cells	Cell free DMSO extracts
Control	30.80	37.69
1 % NaCl	30.06	47.49
2 % NaCl	18.92	38.26
3 % NaCl	40.45	34.94
4 % NaCl	24.86	46.15
5 % NaCl	40.45	40.37
1 mM H_2_O_2_	62.72	44.29
2 mM H_2_O_2_	50.84	34.60
3 mM H_2_O_2_	38.22	33.65
4 mM H_2_O_2_	54.55	50.15
5 mM H_2_O_2_	43.42	29.04

### Radical scavenging capacities

The results of RSC estimations are strongly dependent on the testing system. No single method can assure the complete examination of antioxidant capacity in the samples under investigation. Antioxidant activity may be more reliably assessed by a combination of several tests and assays. In the present study, RSC of extracts was determined using ABTS, DPPH and EPR spin-trapping techniques and correlated with TPC. While ABTS and DPPH tests were used to determine total antioxidant activity, EPR spin-trapping technique was applied to investigate the ability of various DMSO extracts to scavenge the reactive radicals.

**Fig. 1:**
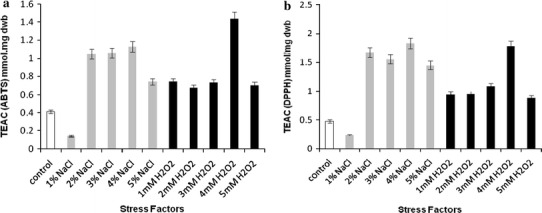
Trolox equivalent antioxidant capacity of DMSO extracts of *R. glutinis* as determined by **a** ABTS assay and **b** DPPH assay

Total antioxidant activity measured by ABTS and DPPH assays were evaluated for extracts obtained from stressed *R. glutinis* and compared with the control. The antioxidant activity established by ABTS test was found to be the highest in culture extracts stressed with 4 mM H_2_O_2_ (1.4 μM TEAC g^−1^) and 4 % NaCl (1.1 μM TEAC g^−1^) as compared to control samples (0.4 μM TEAC g^−1^) (Fig. 1a). Similarly, the total antioxidant capacity as determined by DPPH test was also the highest in cultures stressed with 4 % NaCl (1.8 μM TEAC g^−1^) and 4 mM H_2_O_2_ (1.8 μM TEAC g^−1^) compared to control (0.5 μM TEAC g^−1^) (Fig. 1b). A significant correlation was obtained between total phenolic content vs. ABTS (*R*^*2*^ = 0.9237) and DPPH (*R*^*2*^ = 0.9586) considering the values of 2–5 mM H_2_O_2_. Similarly, a good correlation (the correlation coefficient *R*^*2*^ = 0.8784) was observed between ABTS and DPPH tests. It provides strong evidence that the predominant source of antioxidant activity derives probably from phenolic compounds in yeasts. However, no correlation was observed between phenolic contents from NaCl-stressed cultures and ABTS/DPPH tests. This inconsistency might be due to a change in phenolic profile during cultivation under osmotic stress, for example carotenoids as reported previously (Edge et al. 1997; Marova et al. 2004) might contribute considerably to the antioxidant activity. As stated earlier, stress in biological systems might induce the formation of new metabolites or change the behaviour of organisms under stress. Our results are in conformity with Rapta et al. (2005) who also reported an increased level of total antioxidant capacity of *R. glutinis* stressed with heavy metal ions Ni (II) and Zn (II).

**Fig. 2:**
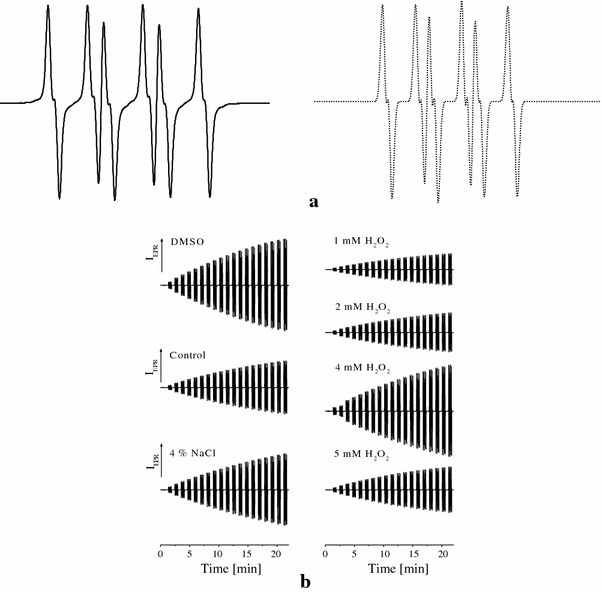
**a** Experimental (*full line*) and simulated (*dotted line*) EPR spectrum (SW = 6 mT) of ^•^DMPO-SO_4_^−^ spin adduct recorded during the thermally initiated decomposition of K_2_S_2_O_8_ in DMSO at 333 K. Simulation spin Hamiltonian parameters: *a*_N_ = 1.296 mT, *a*_H_^β^ = 0.938 mT, *a*_H_^γ^ = 0.139 mT; *g* = 2.0059. **b** Time course of 20 individual EPR spectra obtained for samples of DMSO extracts of NaCl and H_2_O_2_-stressed *Rhodotorula glutinis* CCY 20-2-26. All sets of 20 EPR spectra of DMPO adducts monitored during the thermal (333 K) decomposition of K_2_S_2_O_8_ in the presence of DMSO extracts were taken for 22 min under the same experimental conditions as for reference sample (DMSO, instead of DMSO extracts). Extracts concentrations (μg mL^−1^): control (257), 4 % NaCl (115), 1 mM H_2_O_2_ (207), 2 mM H_2_O_2_ (207), 4 mM H_2_O_2_ (131) and 5 mM H_2_O_2_ (216)

**Fig. 3:**
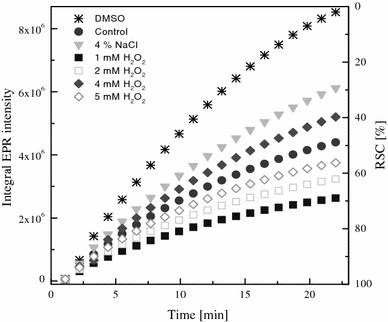
Time course of EPR integral intensities of DMPO adducts (spectra shown in Fig. 2b) recorded during first 22 min of the thermal decomposition of K_2_S_2_O_8_ in the presence of DMSO extracts of *R. glutinis* grown under stress of NaCl or H_2_O_2_

The characteristic experimental EPR spectrum of ^•^DMPO-SO_4_^−^ spin adduct recorded during the thermally initiated decomposition of K_2_S_2_O_8_ in DMSO at 333 K, along with its simulation (*a*_N_ = 1.296 mT, *a*_H_^β^ = 0.938 mT, *a*_H_^γ^ = 0.139 mT; *g* = 2.0059) are shown in Fig. 2a. Figure 2b represents the original sets of 20 individual EPR spectra monitored in the presence of DMPO during heating of K_2_S_2_O_8_ at 333 K for the reference sample DMSO (200 μL DMSO, 25 μL 0.2 M DMPO in DMSO, 25 μL 0.01 M K_2_S_2_O_8_ in DMSO) and for DMSO extracts of *R. glutinis* grown under stress of either NaCl or H_2_O_2_ (200 μl extract in DMSO, instead of DMSO in reference sample). It should be noted here that the decrease in the directly monitored EPR signal intensity of spin adducts is influenced by the concentration of individual extracts (Fig. 2b). Figure 3 shows a time dependence of EPR integral intensities (evaluated by double integration of sets of 20 individual EPR spectra for each measurement, e.g. Fig. 2b) representatively for the extracts of *R. glutinis*. The EPR integral intensity after 22 min detected for the extracts was compared to that of the reference. The difference between these EPR intensities is proportional to the amount of radicals terminated by the scavengers present in the investigated extract sample (Fig. 3), and is called relative radical scavenging capacity (RRSC, expressed in %). Finally, RRSC values were recalculated to the TEAC values (molar amount of trolox/1 g of dry extract inducing the identical changes in RRSC) using calibration curve obtained under strictly identical conditions for trolox solutions in K_2_S_2_O_8_/DMPO/DMSO systems.

**Fig. 4:**
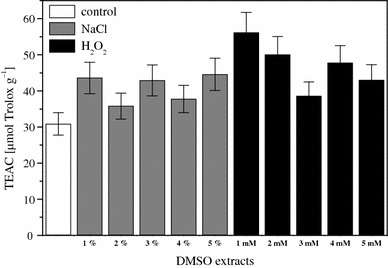
Radical scavenging capacities (equated to actual dry weight of the samples) expressed as TEAC and evaluated by EPR spin trapping of DMSO extracts of *R. glutinis* grown under various concentrations of NaCl or H_2_O_2_

The radical scavenging ability from EPR spin-trapping assay of H_2_O_2_-stressed cultures ranged from 38.6 to 56.1 μmol trolox g^−1^ with the highest in 1 mM H_2_O_2_ (56.1 μmol TEAC g^−1^). Whereas in NaCl-stressed cultures, it ranged from 35.8 to 44.6 μM TEAC g^−1^ with the highest in 5 % NaCl (44.6 μM TEAC g^−1^) as compared to control samples (30.9 μM TEAC g^−1^) (Fig. 4). The EPR spin-trapping and ABTS/DPPH spectrophotometric results are not correlated. This may be explained due to operation of different mechanism of action in the tests used, as most probably distinct reaction pathways are involved in the termination of a stable DPPH and ABTS^•+^ radical species and the reactive SO_4_^•−^ radical anion (Zalibera et al. 2009) by active compounds present in the investigated samples. In EPR experiments, we generate the SO_4_^•−^ with high redox potential, which are capable to react with a variety of organic compounds present in the extracts (e.g. glucan, polysaccharides and carotenoids) other than phenolics. Whereas in DPPH and ABTS assays, the dominating compounds scavenging radical species are H-donor antioxidants. However, in general it was observed that extracts of cultures grown under stress showed higher radical scavenging ability than unstressed cultures. Overall, there was a significant increase in phenolic content and RSC of *R. glutinis* when stressed with NaCl or H_2_O_2_. This is a first report of occurrence of phenolic compounds in *R. glutinis*.

### HPLC determination of phenolic compounds

**Fig. 5:**
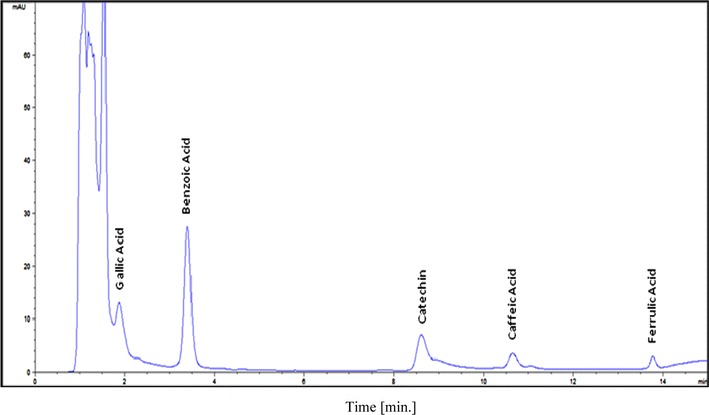
HPLC profile of the DMSO extracts of *R. glutinis* showing presence of various phenolic compounds at 272 nm

The DMSO extracts of *R. glutinis* were evaluated using HPLC. Preliminary studies with mobile phase of acetonitrile and acidified water with acetic acid (pH 2.8) were conducted. The phenolic compounds were detected at 272 nm. All the samples reported positive for various phenolic compounds viz., gallic acid, benzoic acid, catechin, caffeic acid and ferulic acid in the experiential peaks (Fig. 5). It was observed that there is a metabolic shift in cultures when stressed with either NaCl or H_2_O_2_. Using HPLC, Rizzo et al. (2006) determined phenolics adsorbed on yeasts grown on different media. Phenolic compounds produced by sclerotia of the fungus *Inonotus obliquus* have been reported to be the active constituents responsible for antioxidant activities (Zheng et al. 2009). However, there is no report of occurrence of phenolics in yeasts. In the present investigation, extracts prepared from yeasts grown under stress of H_**2**_O_2_ or NaCl reported five peaks indicating a significant amount of phenolic compounds in the investigated species.

## Conclusions

The results of the present study reported that *R. glutinis* when grown under stress of either NaCl or H_2_O_2_ resulted in an increased amount of total phenolic compounds. The results were further supported by the enhanced RSC of the extracts obtained from stressed cultures. A total of five phenolic compounds viz., gallic acid, benzoic acid, catechin, caffeic acid and ferulic acid were detected for the first time in *R. glutinis.* This investigation will further form a basis for the use of yeasts as a source of antioxidants and in formulation of functional foods and pharmaceutical preparation.

## Abbreviations

ABTS: 2,2′-Azino-bis(3-ethylbenzothiazoline-6-sulfonic acid) diammonium salt
DCW: Dry cell weight
DMPO: 5,5-Dimethyl-1-pyrroline *N*-oxide
DMSO: Dimethylsulfoxide
DPPH: 1,1-Diphenyl-2-picrylhydrazyl
EPR: Electron paramagnetic resonance
FC: Folin and Ciocalteu’s phenol reagent
GAE: Gallic acid equivalent
*R*: Correlation coefficient
RRSC: Relative radical scavenging capacity
RSC: Radical scavenging capacity
SW: Magnetic field sweep
TEAC: Trolox equivalent antioxidant capacity
TPC: Total phenolic content
UV–Vis: Ultraviolet/visible
